# Comparison of Different Doses of Pregabalin to Prevent Succinylcholine-Initiated Fasciculations and Myalgia in Patients Under General Anesthesia: A Randomised Controlled Study

**DOI:** 10.7759/cureus.66985

**Published:** 2024-08-16

**Authors:** Malavika Sasidharan, Renuka Holyachi, Pratibha S D

**Affiliations:** 1 Anaesthesiology, Shri B. M. Patil Medical College Hospital and Research Centre, BLDE (Deemed to be University), Vijayapura, IND

**Keywords:** pain, succinylcholine, pregabalin, myalgia, fasciculation

## Abstract

Background

Succinylcholine (SCh) is the most commonly used muscle relaxant during endotracheal intubation, and it is known to cause fasciculations and postoperative myalgia. Pregabalin is structurally similar to the neurotransmitter gamma-aminobutyric acid (GABA), which is known to reduce SCh-induced fasciculations and myalgia.

Materials and methods

This study was conducted on patients who underwent surgery under general anesthesia. A total of 201 patients of both genders were assigned to one of the following groups: Group PL (pregabalin low dose) received cap pregabalin 75 mg, Group PH (pregabalin high dose) received cap pregabalin 150 mg, and Group P (placebo) received saccharine pill 10 mg, administered two hours prior to surgery.

Results

Both the incidence and severity of fasciculations and myalgia were reduced in patients who received pregabalin compared to the placebo group (PH>PL>P). It was observed that as the severity of fasciculations increased, myalgia also increased. The time of the first analgesic dose was longer in the pregabalin group (PH>PL>P). The attenuation of pressor response and hemodynamic stability was greater in the pregabalin group (Group PH>PL>P). Sedation levels were insignificant among groups. The incidence of adverse effects was also insignificant.

Conclusion

Preoperative prophylactic administration of pregabalin orally in Group PL and PH reduced the incidence and severity of fasciculations and myalgia. Group PH was found to be more effective than PL. Pressor response attenuation was found to be more effective in Group PH.

## Introduction

Succinylcholine (SCh) is predominantly used during tracheal intubation in patients posted for surgery under general anesthesia (GA) due to its rapid onset and shorter duration of action. Although Western countries use other muscle relaxants, developing countries such as India routinely use SCh [1]. Since the drug provides better relaxation and intubation conditions, it is considered during laryngoscopy and intubation. It is known to cause side effects such as fasciculations, myalgia, bradycardia, hyperkalemia, and malignant hyperthermia. The most commonly encountered are fasciculations and postoperative myalgia [2]. The resultant myalgia from this stress on the muscle fibers due to fasciculations can last for days to weeks, most commonly seen in areas such as the neck, shoulder, and upper abdominal muscles [3]. Adverse effects such as myalgia are distressing to the patients and can prolong the duration of hospital stay, especially for those patients posted for daycare procedures [4].

Many drugs, such as atracurium, dexmedetomidine, magnesium sulphate, opioids, benzodiazepines, phenytoin, ketorolac, and diclofenac, have been studied to prevent postoperative myalgia and fasciculations. Gabapentin, an analog of pregabalin, has been observed to prevent fasciculations and postoperative myalgia but requires a larger dose of the drug to produce significant results. Pregabalin, an α2δ (alpha 2 delta) calcium channel antagonist, shares a similar structure to that of neurotransmitter gamma-aminobutyric acid (GABA), and it blocks the release of neurotransmitters in the presynaptic neurons [5].

Compared to gabapentin, pregabalin is known to be more effective even at a lower dose, thereby decreasing adverse effects such as drowsiness, and myalgia. Very few similar studies have been done with low doses of pregabalin. Also, pregabalin has almost no drug-drug interactions or enzyme-inhibitory properties, thereby not interfering with the metabolism and excretion of other drugs [6]. In this study, we are comparing higher and lower doses of pregabalin with a control group to determine whether a low dose is adequate to suppress the occurrence of SCh-induced fasciculations and myalgia or if a higher dose is required. Vital parameters were also evaluated to determine the action of pregabalin on pressor response due to tracheal intubation. Pressor response is generally characterised by an increased heart rate and blood pressure. In our study, these responses were also assessed, both with a lower dose and a higher dose, as increased somnolence is said to be a common side effect of the gabapentinoid group of drugs, especially for gabapentin. In our study, we assessed any significant sedation caused by pregabalin, using the Ramsay sedation score.

## Materials and methods

Our study was registered at ClinicalTrials.gov under the registration number CTRI/2023/03/050162. The study was started after obtaining approval from the Institutional Ethical Committee (IEC) - Bijapur Lingayat District Educational (BLDE) Deemed to be University's Shri B. M. Patil Medical College Hospital and Research Centre (approval number: BLDE(DU)/IEC/781/2022-23). The study was conducted in accordance with the ethical standards outlined in the Declaration of Helsinki. A total of 201 patients, including both males and females, who met the criteria of ASA (American Society of Anesthesiologists) physical status I and II, between the ages of 18 and 60, and who were scheduled for elective surgeries under general anesthesia, were included in the study. Patients with drug allergies, seizure history, diabetes mellitus, hypertension, cardiac diseases, impaired kidney or liver function, raised intracranial pressure or intraocular pressure, pregnant and lactating females, those who are on calcium channel blockers, and medications for epilepsy and depression were excluded.

All patients were kept nil per orally for eight hours before surgery. On the day of surgery, patients were supplemented with oral medications two hours before surgery. Patients in Group pregabalin low dose (Group PL) received one capsule of pregabalin 75 mg, Group pregabalin high dose (Group PH) received a capsule of pregabalin 150 mg, and patients in Group placebo (Group P) received a saccharine pill 10 mg. The person who administered these medications was blinded, and observations were done by another person who was also blinded from the study. Patients were shifted to the operating room, and IV fluids (dextrose normal saline) were started. Standard monitors attached to record noninvasive blood pressure, pulse oximetry, echocardiography, and baseline vitals were recorded. All patients were premedicated with ondansetron 4 mg IV, midazolam 1 mg IV, and glycopyrrolate 0.2 mg IV, given analgesia using fentanyl 2 mcg/kg IV and induced with propofol 2 mg/kg IV. SCh, 1.5 mg/kg IV, was administered for tracheal intubation. The incidence and severity of SCh-induced fasciculations were observed and given a score of 0 to 3.

Grade 0 for no fasciculation, Grade 1 for mild (fine movements over the face or fingers), Grade 2 for moderate (on both sides and limbs moving), and Grade 3 for severe (widespread, continuous fasciculation) [6].

Patients were intubated with proper-sized endotracheal tubes, and tube placement was confirmed by auscultation. Anesthesia was maintained using nitrous oxide and oxygen (2:1), isoflurane 0.6%, and atracurium IV at optimal maintenance doses. After the end of surgery, neuromuscular blockade was reversed with neostigmine and glycopyrrolate. The efficacy of attenuating the pressor response was assessed using heart rate (HR) and mean arterial pressure (MAP) recorded before induction (base), during induction, intubation, and after one minute, three minutes, five minutes, 10 minutes, and 15 minutes, respectively. The incidence and severity of myalgia were observed after 24 hours of surgery, and severity was given a score of 0 to 3.

Grade 0 for no muscle pain, Grade 1 for mild (muscular pain in one area and no treatment needed), Grade 2 for moderate (muscular pain present and treatment required), and Grade 3 for severe (generalized, severe pain requiring more treatment) [6].

The patient's requirement and the time of the first dose of postoperative analgesia (injection of diclofenac 75 mg) were recorded. The sedation level of the patient was assessed at two hours and six hours after supplementation of pregabalin or placebo. Sedation was assessed by Ramsay sedation score [7].

Statistical analysis

With anticipated incidence and severity of postoperative myalgia between control and pregabalin with 150 mg, 25.7% and 7.1%, respectively, according to the study by Khan et al. [8], the study required sample size of 67 in each group (i.e., a total sample size of 201 for three groups assuming equal group sizes) to achieve a power of 80% for detecting a difference in proportions between groups at a two-sided p-value (probability value) of 0.05.

The data obtained were entered in an MS Excel sheet (Microsoft Corporation, Redmond, Washington), and statistical analysis was performed using IBM SPSS Statistics for Windows, Version 20 (Released 2011; IBM Corp., Armonk, New York). Results were presented as mean ± standard deviation, median and interquartile range, frequency, percentages, and diagrams. Normally distributed continuous variables between three groups were compared using the ANOVA (Analysis of Variance) test. For not normally distributed variables, the Kruskal-Wallis test was used. Categorical variables between the two groups were compared using the chi-square test. A p-value of <0.05 was considered statistically significant. All statistical tests were performed in two-tailed.

## Results

**Figure 1:**
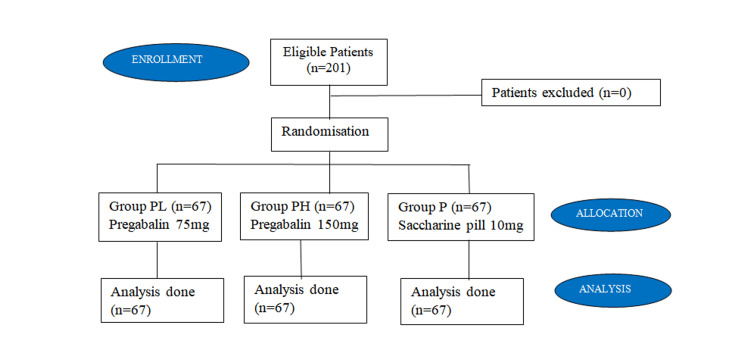
CONSORT flow diagram CONSORT: Consolidated Standards of Reporting Trials; n: number of patients.

Our study incorporated 201 patients, who were randomised into three groups, Group PH, PL, and P, by computer-generated randomisation. We have also followed the CONSORT (Consolidated Standards of Reporting) guidelines in our study (Figure 1).

Demographic data were statistically insignificant. Age at <20, 20-29, 30-39, 40-49, and >50 years and sex (female and male) were represented as numbers (percentages). Age and sex were calculated using the chi-square test. The mean BMI (body mass index) in kg/m^2 ^and duration of surgery in minutes were expressed as mean±standard deviation. Both were calculated using the Kruskal-Wallis test. There was no statistical significance in any of these demographic data (significant p-value set at <0.05). All demographic parameters were comparable between the three groups (Table 1).

**Table 1:** Demographic data Data for age (in years) and sex (female and male) are represented as numbers (percentages), and the mean BMI (in kg/m^2^) and duration of surgery (in hours) are represented as mean±standard deviation. All parameters have a p-value>0.05, indicating that there is no statistical significance. BMI: body mass index.

Parameters	Group PL	Group PH	Group P	p-value
Age (years)				
<20	5(7.5%)	7(10.4%)	7(10.4%)	0.098
20-29	26(38.8%)	10(14.9%)	18(26.9%)	
30-39	13(19.4%)	16(23.9%)	16(23.9%)	
40-49	8(11.9%)	20(29.9%)	14(20.9%)	
>50	15(22.4%)	14(20.9%)	12(17.9%)	
Mean BMI (kg/m^2^)	23.452±2.6365	22.89±1.7556	23.445±1.8114	0.342
Sex				
Female	33(49.3%)	35(52.2%)	33(49.3%)	0.923
Male	34(50.7%)	32(47.8%)	34(50.7%)	
Duration of surgery (hours)	2.6±0.605	2.64±0.62	2.58±0.607	0.846

The incidence of fasciculation and myalgia was 97% and 95.5%, respectively, in Group PL, 5O.7% and 51.7% in Group PH, and 89.6% and 94% in Group P. On analysing this data, the incidence of fasciculation and myalgia has drastically reduced in Group PH, where a higher dose of 150 mg pregabalin was given. A total of 91% of the patients in Group PL had mild to moderate fasciculation, and 89.5% had mild to moderate myalgia, whereas 44.8% of patients in Group PH had only mild fasciculation and myalgia. In Group P, 70.2% of patients had moderate to severe fasciculations, and 68.6% had moderate to severe myalgia. On analysing the severity of fasciculations and myalgia, it was observed that severity was reduced significantly by both doses of pregabalin. However, the patients administered with a higher dose of pregabalin (Group PH) showed very low incidence and severity for fasciculations and myalgia (Table 2).

**Table 2:** Incidence and severity of fasciculations and myalgia *represents statistical significance (level of significance set at p-value<0.05). All data were represented as numbers (percentages).

Parameters	Group PL	Group PH	Group P	p-value
Incidence of fasciculations				
Yes	65(97%)	34(50.7%)	60(89.6%)	<0.001*
No	2(3%)	33(49.3%)	7(10.4%)	
Severity of fasciculations				
Mild	36(53.7%)	30(44.8%)	13(19.4%)	<0.001*
Moderate	25(37.3%)	2(3%)	28(41.8%)	
Severe	4(6%)	2(3%)	19(28.4%)	
Incidence of myalgia				
Yes	64(95.5%)	34(51.7%)	63(94%)	<0.001*
No	3(4.5%)	33(49.3%)	4(6%)	
Severity of myalgia				
Mild	35(52.2%)	30(44.8%)	17(25.4%)	<0.001*
Moderate	25(37.3%)	2(3%)	25(37.3%)	
Severe	4(6%)	2(3%)	21(31.3%)	

For fasciculations, a significant p-value of <0.001 was obtained between both Group PL vs. PH and PH vs. P. In contrast, it was statistically insignificant between Group PL vs. P. Hence, the incidence and severity of fasciculations were significantly less in Group PH. For myalgia, a significant p-value of <0.001 was obtained between all the groups; therefore, it can be concluded that the incidence and severity of myalgia were significantly less in Group PH, followed by Group PL compared to Group P. These data were assessed using the Kruskal-Wallis test. On analysing these data, it was observed that as the dosage of pregabalin increased, the severity of fasciculations and myalgia decreased (Table 3).

**Table 3:** Comparison of incidence and severity of fasciculations and myalgia between groups *represents statistical significance (the level of significance set at p-value<0.05). An intergroup comparison depicting test statistics, standard error, and standard test statistic values using the Kruskal-Wallis test.

Parameters	Test Statistics	Standard Error	Standard Test Statistics	p-value
Fasciculations				
Group PL vs. Group PH	54.187	9.575	5.659	<0.001*
Group PH vs. Group P	-76.627	9.575	-8.003	<0.001*
Group PL vs. Group P	-22.440	9.575	-2.344	0.057
Myalgia				
Group PL vs. Group PH	53.493	9.558	5.597	<0.001*
Group PH vs. Group P	-79.604	9.558	-8.329	<0.001*
Group PL vs. Group P	-26.112	9.558	-2.732	0.019

**Figure 2:**
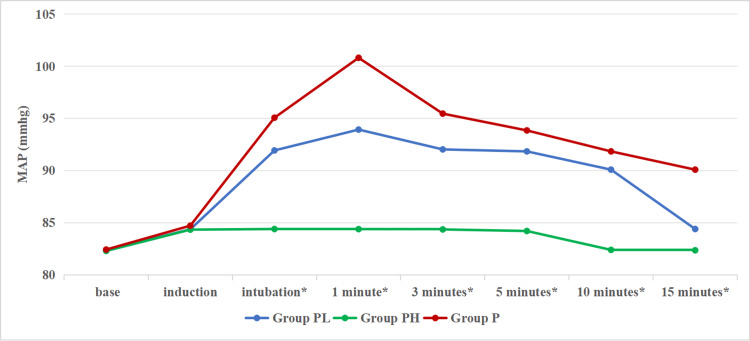
Comparison of mean arterial pressure at baseline, induction, one minute, three minutes, five minutes, 10 minutes, and 15 minutes *represents statistical significance (the level of significance set at p-value<0.05). The p-value at baseline is 0.925, at induction is 0.540, and from intubation until 15 minutes is <0.001. MAP (mmHg): mean arterial pressure (millimeters of mercury).

The MAP was assessed using the Kruskal-Wallis test. Among the Groups PL, PH, and P, a significant p-value was obtained from intubation until 15 minutes. The attenuation of MAP back to baseline was significantly faster in Group PH, followed by Group PL and Group P. Even though the rise in MAP was reduced by both doses of pregabalin, it was maintained near to baseline with a higher dose of pregabalin (Figure 2).

**Figure 3:**
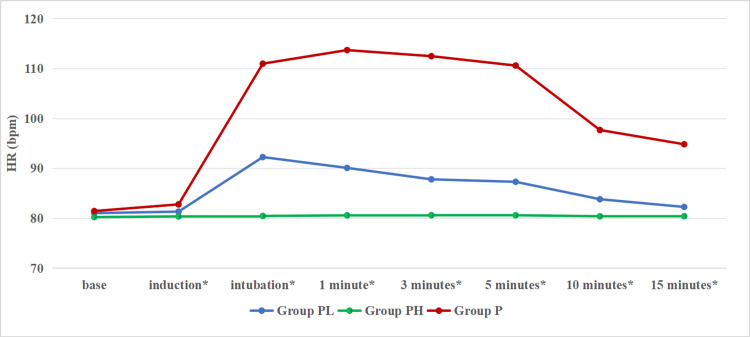
Comparison of heart rate at baseline, induction, one minute, three minutes, five minutes, 10 minutes, and 15 minutes *represents statistical significance (the level of significance set at p-value<0.05). The p-value at baseline is 0.637, at induction is 0.017, and from intubation until 15 minutes is <0.001. HR (bpm): heart rate (beats per minute).

HR was assessed using the Kruskal-Wallis test. Among the Groups PL, PH, and P, a significant p-value was obtained from induction until 15 minutes. The attenuation of MAP back to baseline was significantly faster in Group PH, followed by Group PL and Group P. HR was observed to be maintained near baseline with a higher dose of pregabalin. Although the rise in HR was reduced by both doses of pregabalin, it was maintained near to baseline with a higher dose of pregabalin (Figure 3).

The first analgesic dose was given in Group P followed by Group PL and Group PH after surgery and was statistically significant with a p-value<0.001. The time of the first analgesic dose was lengthened in Group PH.

The mean sedation score at two and six hours among groups was noted using the Ramsay sedation score, and the incidence of adverse effects among groups was assessed, which was found to be statistically insignificant.

## Discussion

SCh is a short-acting depolarising muscle relaxant commonly used for laryngoscopy and tracheal intubation in patients posted for surgery under general anesthesia. Although developed countries, especially Western regions, are using other muscle relaxants, developing countries such as India still consider SCh for laryngoscopy and intubation [9-12]. SCh is known to cause its most typical side effect, fasciculations, which leads to severe postoperative myalgia in these patients. For those who are posted for daycare surgeries, adverse effects, such as myalgia, are very distressing as they can lead to prolonged hospital stays [13-15]. Although self-limiting, in order to reduce the incidence and severity of these side effects, various methods and medications have been studied [6]. Multiple studies have been done regarding the role of drugs such as lidocaine, magnesium sulphate, clonidine, NSAIDs, and NMDRs, such as rocuronium and cisatracurium [16-18]. Most of these drugs helped reduce the severity of fasciculations but not the incidence of fasciculations. Subsequently, the gabapentinoid group (gabapentin and pregabalin) was studied with higher doses for reducing the incidence and severity of fasciculations and was found to be effective [19-21]. However, studies using low doses and large sample sizes were less frequent, especially for pregabalin. Hence, in this study, we compared both low-dose and higher-dose pregabalin with a control group to prevent SCh-induced fasciculations and myalgia [12,15].

Procedures such as laryngoscopy and intubation can cause an exaggerated pressor response that leads to a sudden rise in HR, blood pressure, etc. Drugs such as gabapentinoids are also used to attenuate this response to maintain hemodynamic stability, and pregabalin is known to have fewer side effects, such as sedation. In our study, two doses of pregabalin (75 mg and 150 mg) were compared with a control group to assess the efficacy of attenuating these responses and the effect on SCh-induced fasciculations and myalgia. The gabapentinoid group of drugs are known for its sedative effect, but pregabalin has a lesser sedative effect compared to gabapentin. Side effects such as bradycardia are also seen less with pregabalin [1]. Since the gabapentinoid group is known to reduce fasciculations, myalgia, pressor responses, postoperative analgesic requirements, etc., it makes pregabalin a better choice compared to other drugs [1].

As per the observed results from our study, 75 mg and 150 mg of pregabalin were effective in attenuating both the incidence and severity of fasciculation and myalgia, whereas 150 mg was more effective. In our study, we observed that the pressor response was also attenuated by both doses of pregabalin, where 150 mg was comparatively better. On assessing the time of the first analgesic dose, both dosages prolonged the time of requirement for postoperative analgesia. Sedation levels were not significant in any of the groups. Adverse effects were also very minimal among the groups.

Velez et al. recorded that the incidence of myalgia was significantly reduced in patients who were given gabapentinoid (gabapentin or pregabalin) [3]. In our study, the incidence of myalgia was significantly lower with both a higher dose of 150 mg and a low dose of 75 mg pregabalin compared to placebo. Rashmi et al. demonstrated that low-dose pregabalin, 75 mg, reduced the severity, whereas there was no substantial effect on the incidence of both muscle twitches and muscular pain [7]. In our study, we observed that both doses of pregabalin, 75 mg and 150 mg, were effective in reducing the incidence and severity of fasciculations and myalgia, but more effective with 150 mg. Iqbal et al. conducted a study on preventing fasciculations, myalgia, and hyperkalemia due to SCh in patients posted for spine surgery [6]. One group received oral pregabalin 150 mg, and the other received a placebo one hour before surgery. It was observed that the severity of fasciculations, the incidence and severity of myalgia, and serum potassium levels were reduced by pregabalin. Total opioid consumption was also found to be reduced in the pregabalin group. Our study showed that both the incidence and severity of fasciculations and myalgia were reduced with pregabalin 150 mg, and the first onset of the analgesic requirement was delayed in the postoperative period.

Khan et al. demonstrated that the intensity of muscle twitch was reduced in patients who took pregabalin 150 mg, whereas both the incidence and severity of myalgia were reduced in the pregabalin 150 mg group [8]. Our study observed that both the incidence and severity of fasciculations were reduced with pregabalin, even with a low dose of 75 mg, but 150 mg was comparatively better. Shrivastava et al. conducted a study, in which one group received pregabalin 150 mg, and the other group received a placebo one hour before the induction of anesthesia [2]. It was observed that the incidence of fasciculations was not significant in both groups, whereas the severity was moderate to severe in the placebo group. Both the incidence and severity of myalgia were low in the pregabalin group. In our study, it was observed that both the incidence and severity of fasciculations were reduced with pregabalin, even with a low dose of 75 mg but comparatively better was 150 mg.

Parveen et al. conducted a study with oral clonidine 0.3 mg and pregabalin 150 mg administered 60 minutes prior to surgery for attenuation of pressor response in 80 patients posted for laparoscopic cholecystectomy. Clonidine and pregabalin were observed to reduce these responses, with clonidine being more effective but also causing more bradycardia [21]. In our study, none of the patients, both low-dose and high-dose pregabalin, show any bradycardia, making it more hemodynamically stable. Jain et al. demonstrated in their study that the group who received pregabalin 75 mg had less postoperative pain and lesser requirement for other analgesics than the placebo group [12]. In our study, the onset of the first rescue analgesia was also delayed in patients who received even a low dose of 75 mg pregabalin. Rastogi et al. demonstrated that MAP was significantly attenuated with pregabalin 150 mg and no significant change in any other group [1]. In our study, there was significant attenuation of MAP and HR even in patients who received a low dose of 75 mg of pregabalin, but comparatively more attenuation happened with 150 mg of pregabalin. Sedation was recorded using Ramsay sedation score.

Limitation

The limitations of our study were that only patients classified as ASA I and II were included, serum potassium levels were not assessed, and the study was limited to our institution. Caution should be exercised while delivering pregabalin to patients classified as ASA III and IV. SCh can cause adverse effects such as hyperkalemia, therefore, it is advisable to assess serum potassium levels in patients with comorbidities. Electromyography (EMG) was not used to assess fasciculation. In order to consider pregabalin in standard guidelines, studies involving multiple institutions might be needed.

## Conclusions

Preoperative prophylactic administration of oral pregabalin at 75 mg and 150 mg reduced the incidence and severity of SCh-induced fasciculations and myalgia. A dose of 150 mg was found to be more effective than 75 mg. Side effects were not significant at a dose of 150 mg. Pressor response attenuation was found to be more effective at a dose of 150 mg compared to 75 mg. Hemodynamic stability was maintained at both pregabalin doses. SCh continues to be a muscle relaxant of choice for intubation in cases of anticipated difficult airways. Prophylactic pregabalin can be used to reduce myalgia in the postoperative period. Therefore, we conclude that preoperative oral pregabalin is an effective and safe method for the prevention of SCh-induced fasciculations and myalgia.
